# Contaminants of Emerging Concern: Antibiotics Research in Mussels from the Coasts of the Tyrrhenian Sea (Sardinia, Italy)

**DOI:** 10.3390/ani14081205

**Published:** 2024-04-17

**Authors:** Filomena Dessì, Maria Vittoria Varoni, Elena Baralla, Maria Nieddu, Valeria Pasciu, Gabriella Piras, Giuseppa Lorenzoni, Maria Piera Demontis

**Affiliations:** 1 Department of Veterinary Medicine, University of Sassari, 07100 Sassari, Italy; f.dessi@studenti.uniss.it (F.D.); varoni@uniss.it (M.V.V.); vpasciu@uniss.it (V.P.); dpiera@uniss.it (M.P.D.); 2 Department of Medicine Surgery and Pharmacy, University of Sassari, 07100 Sassari, Italy; marvi@uniss.it; 3 Veterinary Public Health Institute of Sardinia, 07100 Sassari, Italy; gabriella.piras@izs-sardegna.it (G.P.); pina.lorenzoni@izs-sardegna.it (G.L.)

**Keywords:** emerging contaminants, macrolides, bivalves, animal models, LC-MS/MS, marine pollution

## Abstract

**Simple Summary:**

Antibiotics are emerging contaminants found in several matrices of environmental, animal, and human origin. This high occurrence constitutes a major global threat, which is also responsible for the development of antimicrobial resistance. Therefore, it is vitally important to develop new, simple, and reliable methods by which to quantify them in several matrices. In this work, the mussel matrix was chosen as the sentinel organism; a new analytical method for the determination of two commonly used macrolides in mussels harvested along the Sardinian coasts was developed. None of the tested macrolides were found in the sampling sites at concentrations quantifiable by the developed method. These results can be useful for competent authorities evaluating the food safety of mussels (as concerns the studied antibiotics) and pollution derived from these drugs in the marine environment.

**Abstract:**

Contaminants of emerging concern (CECs) are compounds found in several environmental compartments whose ubiquitous presence can cause toxicity for the entire ecosystem. Several personal care products, including antibiotics, have entered this group of compounds, constituting a major global threat. It is essential to develop simple and reliable methods by which to quantify these contaminants in several matrices. In this work, mussels were chosen as sentinel organisms to assess environmental pollution and the safety of bivalve mollusk consumption according to the “One Health perspective”. A liquid chromatographic tandem mass spectrometry method (LC-MS/MS) was developed for the quantification of two macrolides, erythromycin (ERY) and azithromycin (AZI), in mussels. This new method was validated according to international guidelines, showing high selectivity, good recoveries (>60% for both of them), sensitivity, and precision. The method was successfully applied for ERY and AZI research in mussels farmed along the Sardinian coasts (Italy), demonstrating itself to be useful for routine analysis by competent authorities. The tested macrolides were not determined in the analyzed sites at concentrations above the limits of detection (LODs). These results demonstrate the food safety of mussels (as concerns the studied antibiotics) and a negligible amount of pollution derived from these drugs in the studied area.

## 1. Introduction

Antibiotics are lifesaver drugs, commonly used in both human and veterinary medicine. They are considered the most frequently used in the world, together with anti-inflammatory drugs [1]. 

An increase of 46% in their global consumption has been noted from 2000 to 2018 [2]. This trend is predicted to increase further in the coming decades, mainly due to the high demand for these drugs in countries such as South Africa, China, Brazil, India, and Russia [3]. 

The high use of these drugs led to the development of the “Silent Pandemic”, namely, antimicrobial resistance (AMR). AMR is a phenomenon that emerged as one of the principal public health problems of the 21st century, but it already existed before the discovery of antibiotics; a microorganism can be insensitive to some antibiotics by nature (i.e., innate resistance). However, acquired resistance represents the real problem for global health. In this latter case, a microorganism which was previously sensitive to an antibiotic develops resistance mechanisms only subsequently, modifying its genetic material. AMR is going to be the main cause of death in the world by 2050 if actions to combat this are not carried out. Several governments are developing strategies to curb this emergency, using the one-health approach, which requires collaborative efforts from different institutes and disciplines [4]. 

In light of this, the World Health Organization (WHO), the European Union (EU), and the United States (USA) have introduced restrictions on antibiotics use in animals and banned them for use in growth-promoting systems. Nevertheless, it is difficult to globally monitor antibiotic use considering the high demand for food of animal origin [5]. 

Moreover, antibiotics are also used in aquaculture in different amounts depending on the country taken into consideration. As an example, antibiotics use in Norway is around 1 g per metric ton, while in Vietnam, it reaches 700 g per metric ton. These high differences are strongly correlated with the restrictions taken by different countries’ governments [6,7]. To face this topic of high concern, and to protect the environment, animals, and consumers’ health, from their “One Health perspective”, the Codex Alimentarius, the European Commission, and the Food and Drug Administration (FDA) introduced the maximum residue limits (MRLs) of approved veterinary drugs in food of animal origin [8]. Unfortunately, MRL values have not been harmonized worldwide, and most developing countries do not have established MRLs yet [7].

Given their excessive use, antibiotics are now considered to be among the contaminants of emerging concern (CECs) because they can enter the environment through multiple sources [9]. Their main sources of release into the environment are wastewater treatment plants (WWTPs), industrial and hospital wastes, aquaculture plants, animal breeding, and soil runoff. WWTPs are not always effective in removing these pharmaceuticals, so a high concentration of drugs can be discharged into the environment [10]. This inadequate removal of antibiotics can result in long-term human and animal exposure to their residues, resulting, in addition to AMR, in adverse biological effects [11,12]. As an example, the accumulation of CECs in aquatic organisms can be responsible for their introduction into the human food chain through seafood consumption [13].

For this reason, proper legislation should be accompanied by monitoring programs able to investigate antibiotics’ presence in environmental compartments. Thus, adequate model organisms should be chosen to assess the risk of antibiotic accumulation for the entire ecosystem [14]. Bivalve mollusks, such as mussels, are often used as sentinel organisms to assess environmental pollution because of their characteristics. They are indeed robust organisms of medium size, easy to breed, and able to survive in adverse conditions. Moreover, they are sedentary, so they are used to investigate the quality of their surrounding environment. Furthermore, mussels filter high volumes of water, retaining the 90% of particles contained in it, including CECs as antibiotics [14,15]. As mussels have a limited capacity to bio-transform environmental contaminants, they are often used as animal models in studies of pollutants’ bioconcentration, bioaccumulation, and toxicokinetic analysis. Furthermore, mussels are ecologically important and are a source of food for several other species, so anthropogenic pollutants can be conveyed into the food chain. Mussels are also used as animal models for aquatic toxicology studies of a wide number of contaminants, like heavy metals, pesticides, and microplastics [16,17,18].

To monitor chemical contaminants in sediments and bivalve shellfish from different coastal zones, plans called “Mussels Watch Programs” have been developed in the USA since the 1970s [19]. Also, France and other countries have begun to plan mussel watch programs, so they may be implemented worldwide to assess the magnitude and distribution of pollutants [20].

In addition, the European Commission has established a list of potential water pollutants (watch list) that should be carefully monitored by the EU Member States to determine the risk they pose to the aquatic environment and to human health. The first watch list was created in 2015 [21] and is updated every two years in order to collect high-quality monitoring data to support the risk-based assessment of CECs. In this list, several antibiotics, including macrolides, are reported. This inclusion is consistent with the European One Health Action Plan against AMR, which supports the use of the watch list to “improve knowledge of the occurrence and spread of antimicrobials in the environment”. 

Macrolides are commonly prescribed antibiotics both in human and veterinary fields, and they represented 7% of total antibiotics use in USA in 2020 [22]. They are bacteriostatic antibiotics that exert their action through their binding with the bacterial 50s ribosomal subunit, causing the inhibition of bacterial protein synthesis.

The first-discovered macrolide was erythromycin (ERY), which has been largely used to treat bacterial infections since 1950s. After this first generation, macrolides of the second generation were developed, exhibiting improved pharmacological properties [23]. Between them, azithromycin (AZI) is a semi-synthetic derivative of ERY with a 15-membered lactone ring with a methyl-substituted nitrogen atom incorporated. This chemical modification makes AZI exhibit broader spectrum activity, longer half-life, and fewer short-term side effects when compared to other antibiotics [24]. Moreover, it has been used to treat a multitude of diseases, like respiratory, dermal, urogenital, and other bacterial infections, and it has been found to be effective against some chronic inflammatory disorders like bronchiolitis or sexually transmitted infections [25,26]. Given these premises, the WHO included it as one of the safest drugs for any national health system [27]. AZI has also been a drug candidate used to treat the global health crisis of COVID-19. It is annually prescribed to more than 40 million patients and this increased the global AMR of AZI [28]. Moreover, AZI mass distribution is sometimes recommended in trachoma control programs in countries with high child mortality [29]. However, an increase in macrolides resistance determinants was found in the gut of treated communities, sometimes associated with a co-resistance also to unrelated antibiotics classes like beta-lactams, aminoglycosides, metronidazole, and trimethoprim. Thus, resistance surveillance plans should always be introduced when developing mass drug distribution programs [30]. The two macrolides, ERY and AZI, have been introduced in the EU watch list because of their potential to determine AMR, and several research papers focus on the risk for AMR after macrolides assumption [31,32,33]. One of the most important mechanisms of antibiotic resistance observed in macrolides is the methylation of an adenine residue in the antibiotic binding site, which precludes its binding [23].

Legal requirements need to be supported by proper analytical methods able to ensure efficient control patterns. Several methods are reported in the literature for the screening and quantification of CECs in environmental matrices and food of animal origin [34,35,36,37,38,39,40]. 

Given the complexity of the mussel matrix, which has a high protein and lipid content, the development of a clean-up step is essential before the subsequent analytical technique. Liquid extraction procedures, together with solid-phase extraction processes, are usually carried out to purify the sample before the analysis. 

The development of a sensible and sensitive method should then be necessary to identify and quantify the analyte of interest, even when it is present in small traces. For this reason, the developed method should have a low limit of detection (LOD) and quantification (LOQ). 

Most of the analytical methods use the liquid chromatography technique interfaced with tandem mass spectrometry detection (LC-MS/MS) for antibiotics quantification. LC-MS/MS, in fact, constitutes the best confirmatory method for the determination of drug residues in food of animal origin [14,38,41]. 

This work has a double aim: on the one hand, to develop a simple and reliable method of quantifying two commonly used macrolides in mussels; on the other hand, to apply the new method for the investigation of the eventual presence of ERY and AZI in mussels (*Mytilus galloprovincialis*) harvested in Italy, along the Sardinian coastline, where over 13,000 tons of mussels per year are produced [42].

## 2. Materials and Methods

### 2.1. Chemicals and Reagents

ERY, AZI, erythromycin-(N-methyl-13C,d3) (IS, internal standard), EDTA, and formic acid were purchased from Merck Life Science S.r.l. (Milano, Italy). Stock standard solutions (1 mg/mL) of antibiotics and IS were prepared in methanol and stored at −20 °C. All proper dilutions were made in the mobile phase. The solvents used were of the highest commercial quality and were obtained from Fluka (Buchs, Switzerland). Ultrapure water was obtained using a Milli-Q Lab water system (Sartorius, Goettingen, Germany).

**Figure 1:**
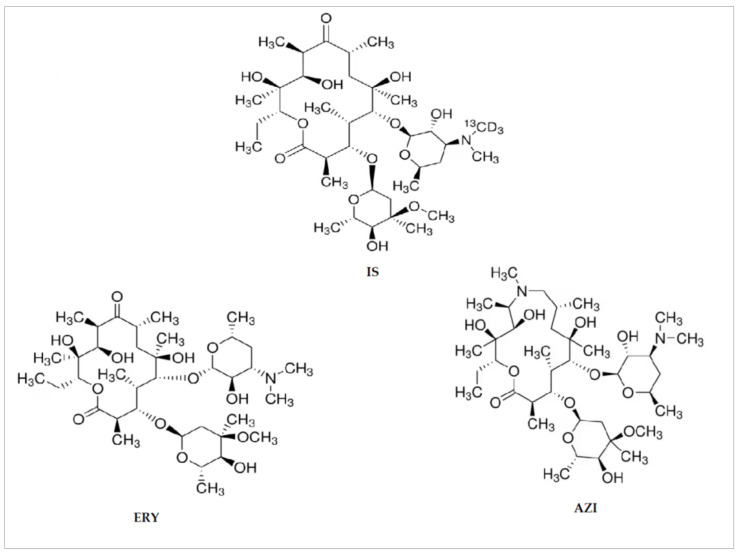
Chemical structure of the antibiotics analyzed in this study. ERY: erythromycin; AZI: azithromycin; IS: internal standard.

The chemical structure of the macrolides analyzed in this study and of the IS are reported in Figure 1. 

### 2.2. Sample Collection and Study Area

**Figure 2:**
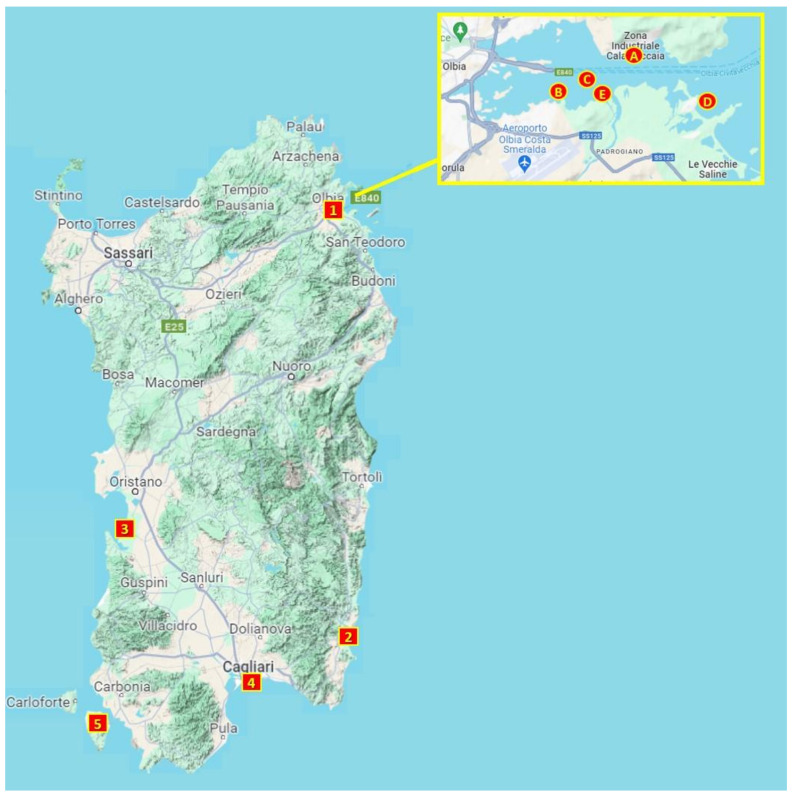
Mussels’ sites collection: 1. Gulf of Olbia: Cala Saccaia (A); Isola del cavallo (B); Mezzocammino rocks (C); Lido del sole (D); Padrongianus (E); 2. Muravera; 3. Arborea; 4. Santa Gilla; 5. Sant’Antioco.

Mussels (*M. galloprovincialis*) were collected in the summer and autumn of 2023 from nine sampling sites in Sardinia (Italy), which has a high impact from a productivity and touristic perspective. Sampling sites belong to production areas located along the island’s coastline (Figure 2). Five of them were located in north Sardinia (Gulf of Olbia: Cala Saccaia 1A, Isola del Cavallo 1B, Mezzocammino rocks 1C, Lido del sole 1D, Padrongianus 1E), one in the center of Sardinia (Arborea: 3), and the other three in south Sardinia (Muravera: 2; Santa Gilla: 4; Sant’Antioco: 5). The Gulf of Olbia, the Gulf of Oristano (Arborea) and the Santa Gilla Lagoon are the main locations of intensive mollusks farms in Sardinia [43]. Moreover, Sant’Antioco Island and Muravera also host important mollusks farms and were chosen in this study to generate a larger distribution of sampling sites.

From each site, a total of 30 mussels were collected as a part of an official control program that included microbiological analysis for classification and monitoring of the production areas. Sampling was carried out at an average depth of 5 m. Mussels (mean length, width, and weight: 7.5, 3.7 cm, and 29.7 g *w*/*w*, respectively) were shelled, and all soft tissues were homogenized using an electric homogenizer to obtain a pool that was then frozen at −20 °C till the preparation for the analysis.

### 2.3. Sample Extraction

After thawing, 0.5 g (wet weight, *w*/*w*) of homogenate was added to 50 µL of EDTA 0.1 M and 2 mL of 0.1% formic acid in acetonitrile. Samples were vortexed for 30 s and shaken for another 10 min. Samples were then centrifuged at 2500× *g* at 4 °C for 10 min. The supernatant was collected into a clean polypropylene tube and dried under a nitrogen stream. 

The dry extract was then reconstituted with the mobile phase containing the IS at a concentration of 200 ng/mL.

### 2.4. LC-MS/MS Analysis

The analysis was carried out using an HPLC ProStar 300 (Varian, Palo Alto, CA, USA) instrument connected to a Varian 310 triple quadrupole mass spectrometer (Varian, Palo Alto, CA, USA). The chromatographic process was carried out on a Phenomenex Luna C18 column (5 µm, 100 × 2.0 mm, Phenomenex, Torrance, CA, USA) fitted with a C18 security guard cartridge (4 × 2 mmID) using a 5 µL loop. A linear gradient with 0.1% formic acid in water (solvent A) and 0.1% formic acid in acetonitrile (solvent B) was performed as follows: 1 min at 5% B; in 29 sec, solvent B was increased to 95%. Then, at 1 min, solvent B decreased to 80% and after 1 sec. The initial conditions (5% solvent B) were restored and maintained for another 5 min and 30 sec. The total run time was 8 min, with a flow rate of 0.2 mL/min. The mass spectrometer was operated in positive electrospray ionization mode (ESI+). Detection conditions for each analyte were first optimized through a direct infusion of standard solutions in the mobile phase with 0.1% formic acid at a concentration of 500 ng/mL. The following ESI source conditions were used: capillary voltage, 65 V; drying gas temperature, 200 °C; nebulizing gas pressure, 50 psi (high-purity nitrogen constituted both nebulizer and drying gas). The collision gas was argon with a pressure of 2 mTorr. The detector voltage was set to 1750 V. The multiple reaction monitoring (MRM) mode was used, and collision energies were optimized for each antibiotic and IS.

Two transitions were considered for each analyte, and, between them, the most abundant one was used for the analyte quantitation, while the other one was used to confirm its identity. Optimized instrumental parameters are reported in Table 1.

Calibration curves were prepared in a blank mussel matrix for each analyte using erythromycin-(N-methyl-13C,d3) as IS. The calibration ranges were from 75 ng/g to 1000 ng/g for AZI and from 25 ng/g to 1000 ng/g for ERY. 

### 2.5. Method Validation

The analytical method was validated according to international guidelines for the validation of analytical methods [44,45]. The parameters analyzed for the investigated pharmaceuticals were selectivity, linearity, LOD, LOQ, precision, recovery, and matrix effect. The selectivity, defined as the ability to quantify the analyte without interferences, was assessed through the analysis of ten extracted blank samples. The linearity of the method was determined using the calibration curves built, using the internal standard method, by spiking matrix extracts with known concentrations of the antibiotics. The LOD and LOQ were determined by calculating the signal-to-noise ratio (S/N) multiplied by 3 and 10, respectively. The precision of the method, expressed as relative standard deviation (RSD), was evaluated via the intra-day and the inter-day repeatability calculated by analyzing the mussel matrix spiked with three concentrations in the expected range (ERY: 100, 200, and 1000 ng/g; AZI: 200, 500, and 1000 ng/g) and extracting six times on the same day for four consecutive days, respectively. The three concentrations (low, medium, and high) used for the evaluation of the method’s precision were chosen considering the linearity calibration range and the sensitivity limits of the two macrolides. The recovery of the method was evaluated at three concentrations for both antibiotics (at 100, 200, and 1000 ng/g for ERY; and at 200, 500, and 1000 ng/g for AZI). For this purpose, blank mussel samples were spiked with the standard antibiotic solutions and extracted as described above. After the analysis, they were compared with the same concentrations of antibiotic standard solutions, which represent 100% of the recovery. Finally, the matrix effect (ME) was evaluated given that this factor can interfere with the analysis through an increase (ion enhancement) or a decrease (ion suppression) in the signal intensity. The matrix effect was determined according to the following:

ME% = [(RM − RS)/RS] × 100,

where RM is the mean peak area of the mussel extract reconstituted with 100, 200, and 1000 ng/g of ERY and with 200, 500, and 1000 ng/g of AZI. RS is the mean peak area of the reference solution at the same concentrations.

### 2.6. Statistical Analysis

Data were analyzed using GraphPad Prism 6 software (GraphPad Software, Inc., La Jolla, CA, USA). 

## 3. Results and Discussion

Several methods are reported in the literature for the analysis of antibiotics in food, environmental, and biological matrices [36,46,47,48,49,50]. However, the main challenge is the creation of a multiresidue method which is able to analyze several antibiotics in a single run. The first hurdle to overcome is related to the ability to isolate the analyte from the matrix. This is particularly true for mussels, a complex matrix with a high lipids and protein content. 

### 3.1. Method Validation

The developed method permitted the identification and quantification of the two macrolides, ERY and AZI, in a single run. Several extraction procedures were tried in order to obtain a fast and effective method able to isolate the analyte from interferences. The extraction procedure used in this work was easy and fast, consisting of a simple liquid extraction, without the requirement for long and expensive equipment and procedures. 

The developed method was demonstrated to be highly selective, with no interferences in selected transitions at the same analytes’ retention time. Calibration response showed good linearity, with a determination coefficient >0.99 for both antibiotics. As regards sensitivity, the LODs and LOQs were 9 and 30 ng/g for ERY and 27 and 90 ng/g for AZI, respectively. The intra-day and inter-day repeatability values, expressed as RSD%, were below 15% for both antibiotics at the three tested concentrations, as required by the international guidelines [44]. Moreover, recovery stood at values higher than 60%. Matrix effect values ranged from values between −27.27 and 27.84 for ERY and between −27.15 and −0.36 for AZI (Table 2).

**Figure 3:**
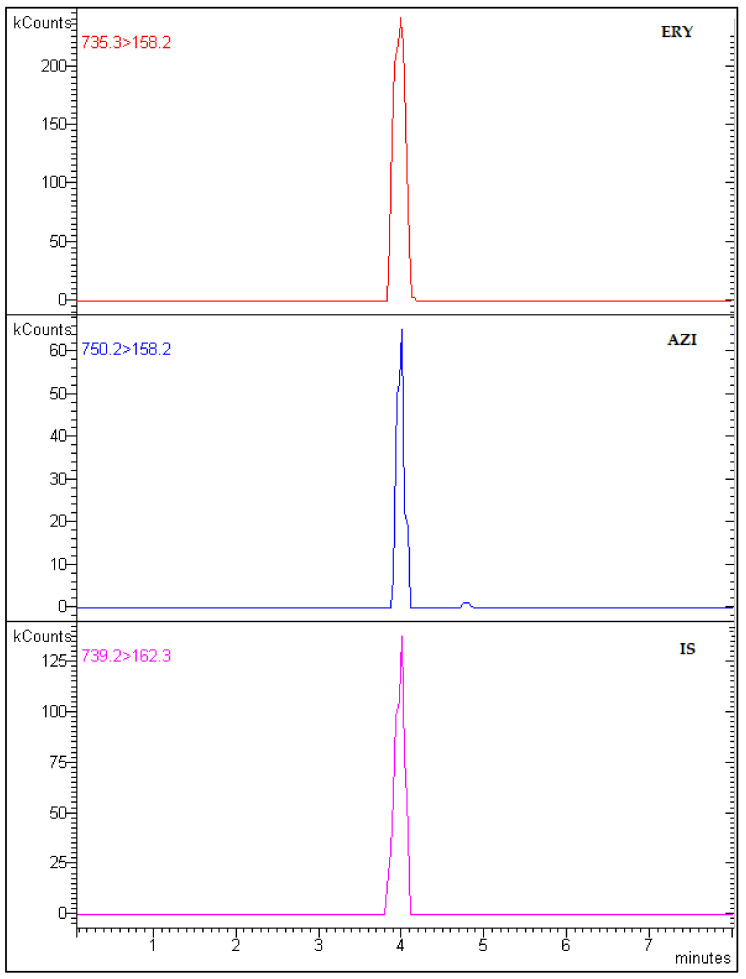
Chromatogram of a drug-free mussel spiked with 200 ng/g of ERY, AZI, and IS.

A chromatogram of a drug-free mussel sample spiked with known concentrations of ERY, AZI, and IS is shown in Figure 3.

### 3.2. Application to Real Samples

The developed and validated method was applied for the research of ERY and AZI in mussels farmed and harvested in Italy, along the Sardinian coasts. It is well known that Italy is among the largest producers of bivalve mollusks in the EU, outranked only to France and Spain. Many studies in the literature have reported on mussels’ safety in order to control various diseases that can be conveyed to humans through their consumption [51,52,53]. The majority of them are focused on the analysis of microbiological parameters or marine biotoxins [54,55,56,57,58,59,60]. Recently, several studies have extended their focus to emerging chemical contaminants or microplastics [45,46,47,48,49,50,51]. The same trend has been observed and reported for the island of Sardinia [59,61,62,63].

Sardinia is one of the main producers of shellfish aquaculture in Italy, with a large production of Mediterranean mussels [58,64]. In this isle, intensive bivalve mollusk farming constitutes 83% of the regional aquaculture production, with *M. galloprovincialis* and *Crassostrea gigas* being the most valuable shellfish species [61].

**Figure 4:**
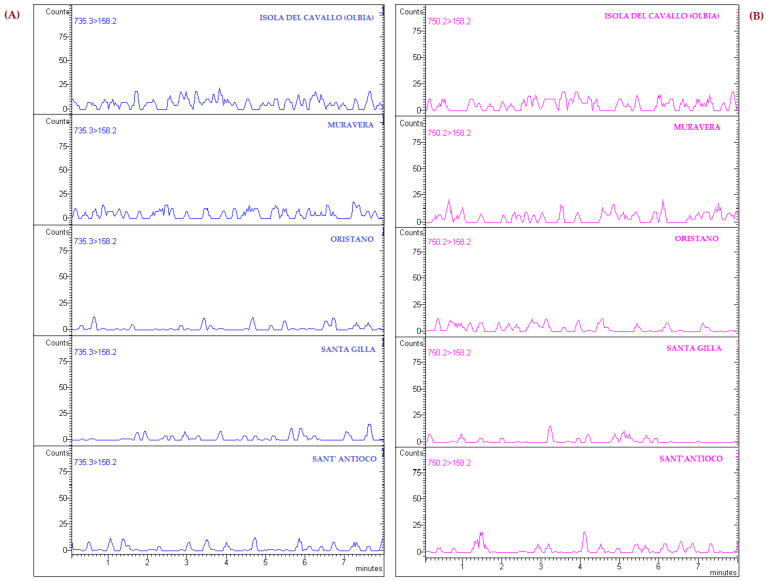
Chromatograms of mussels harvested in the five sample collection sites in Sardinia: (**A**) ERY main transition; (**B**) AZI main transition.

A periodic sanitary control plan for the production and marketing of mollusks has been instituted on Sardinia’s coastline since 1988 to monitor the safety of mollusks during the different stages in the shellfish supply chain [60]. None of the tested antibiotics were found in the analyzed sites at concentrations above the LODs of the method (Figure 4).

Nevertheless, the method described in this work was revealed to be fit for the purpose of quantifying ERY and AZI in the mussel matrix. In fact, considering that no MRL exists for antibiotic residues in mussels, generally, the MRL value assessed for the fish matrix is also applied to mollusks. The MRL value reported for ERY in fish, according to European Union legislation, is 200 ng/g [65], far above the LOD of the developed method. Moreover, despite its large use both in the human and veterinary fields, no MRL has been introduced for AZI in fish, yet. 

Macrolides occurrence has been reported in mussels in different countries—especially in China, which is the world’s largest producer and consumer of antibiotics [66,67,68]. In a previous work, we reported several studies on antibiotics’ presence in bivalves collected all around the world, finding that macrolides represent the most frequently detected classes of antibiotics, followed by sulfonamides and quinolones [14]. 

A recent work reported ERY and AZI concentrations in marine species (fish, shrimps, crabs, and mollusks) between 0.52 and 45.2 ng/g *w/w* in South China. Moreover, AZI and ERY were determined with very high detection frequencies (100% and 87% for AZI and ERY, respectively) [68]. 

This trend is probably linked to the high demand for high-protein foods and to intensive aquaculture programs wherein several antibiotics (including macrolides) are used [69].

Macrolides’ occurrence in aquatic environments has highlighted the need for stronger environmental monitoring programs in order to reduce the toxicological implications for humans, animals, and the environment. Together with the global threat of AMR, macrolides have been found responsible for toxicity to aquatic organisms, resulting in high ecological risk [69].

Nevertheless, in the literature, macrolides’ concentrations in mussels are, to date, all under the MRLs as established by international regulations for the fish matrix [14,65]. The results obtained in the present study indicate that there is no risk, with regard to the antibiotics we researched, to the consumer of mussels collected from the reported sites. Furthermore, given the lack of detection, there is currently no potential risk to the environment from contamination with these two antibiotics. 

However, in a toxicological residues study, the cumulative effect must also be taken into consideration. In fact, humans and animals could be exposed to different compounds that can be simultaneously present in the environment; so, low concentrations should also be considered when referring to the cumulative risk. Moreover, long-term exposure to antibiotics trace levels can also lead to AMR and important changes in gut microbiota [70]. 

Therefore, it is very important to develop new analytical methods that can detect antibiotics in different matrices and, in particular, in sentinel organisms such as mussels. In fact, the matrix chosen in this work has a twofold function: on the one hand, it plays the role of an environmental health indicator; and on the other hand, being a human food, it allows for the food safety assessment of the possible presence of CECs in this matrix.

## 4. Conclusions

This work describes a validated method for the determination in mussels of two macrolides commonly used in human and veterinary medicine.

To our knowledge, this is the first study that investigates the possibility of antibiotics being present in mussels harvested along Sardinia’s coasts. The developed method could be used by competent authorities to assess the safety of bivalve mollusks consumption as concerns the emerging contaminants taken into consideration in this work. Moreover, it will help to evaluate environmental pollution and monitor the wellness of aquatic species. Also, if its sensitivity in terms of the LOD and LOQ is not very high, and recoveries range between 60.5 and 80.01%, this method is still useful for routine controls given that the LOQ is far above the MRL established for ERY in fish and that no MRL exists for AZI yet. Considering mussels’ ability to be used as sentinel organisms, the results obtained in this work also demonstrate that environmental pollution by the research contaminants does not exist in the studied area at the present time. Nevertheless, given antibiotics’ occurrence in several matrices all around the world, monitoring programs including macrolides should be planned by competent authorities. Moreover, new efforts should be directed at establishing the MRLs for CECs in bivalves instead of continuing to use those from fish.

## Figures and Tables

**Table 1:** Instrumental parameters.

Analyte	Precursor Ion (*m*/*z*)	Product Ion (*m/z*)	Capillary (V)	Detector (V)	CE (V)
ERY	735.3	158.2 ^a^ 577.5	65	1750	29 19
AZI	750.2	158.2 ^a^ 592.1	65	1750	28 36
IS	739.2	162.3 ^a^ 83.3	65	1750	28 18

CE: collision energy; ^a^ main transition used for quantitation.

**Table 2:** Precision, recovery, and matrix effect.

Antibiotic	Concentration (ng/g)	Repeatability (RSD %)		Recovery (%) ± SD	Matrix Effect (%)
		Intra-Day	Inter-Day		
ERY	100 200 1000	8.11 10.97 14.75	13.92 12.51 11.89	67.12 ± 5.03 73.55 ± 2.61 62.93 ± 2.92	15.55 −27.27 27.84
AZI	200 500 1000	14.05 7.76 9.47	14.37 14.93 14.7	83.09 ± 3.08 60.53 ± 0.03 63.69 ± 3.43	−20 −0.36 −27.15

RSD%  =  relative standard deviation; SD = standard deviation.

## Data Availability

The data presented in this study are available on reasonable request from the corresponding author.
